# Protein C and S levels in patients with Thalassemia intermedia

**DOI:** 10.25122/jml-2021-0316

**Published:** 2022-11

**Authors:** Nawsherwan Sadiq Mohammed

**Affiliations:** 1 Pathology Department, Hawler Medical University, Kurdistan, Iraq

**Keywords:** protein S, protein C, thalassemia, thromboembolic events

## Abstract

This study was conducted to assess the level of proteins C and S in patients with thalassemia intermedia from the Thalassemia Center in Erbil, Iraq. This study aimed to evaluate protein C and S levels in patients with β-thalassemia intermedia and correlate them to different clinical and laboratory parameters. This comprehensive descriptive case-control study was conducted in 2021. Twenty-three thalassemia intermedia patients were recruited. After the participants' demographic data were recorded, plasma levels of both proteins were measured. The acquired files were examined for the 23 patients studied, 48% of whom were female. The mean age of the patients was 16.32 years. The findings show that the proportion of protein C in males was greater than in females, while this percentage contrasts when compared with protein S (ranging between 89–99% and 85–96%, respectively). Concerning age, these two types of protein in children have more value compared to older ages. Only seven people had less than 1,000 ferritins, while the others had higher values. A decrease in proteins C and S was observed in the thalassemia intermediate compared to the control group. There was a significant relationship between the decreased protein C and S levels with splenectomy. Given the significant reduction in protein C and S levels among patients with thalassemia intermediate compared to the control group, there is an increased risk of thromboembolic events in patients with thalassemia intermediate.

## INTRODUCTION

Thalassemia is a calculated deficiency in the synthesis of globin chains. The disease is common in many regions of the world [1]. Homozygous beta thalassemia generally presents as chronic hemolytic anemia, like major thalassemia, where patients require regular blood transfusions to survive. In contrast, there is no need for regular blood transfusions in thalassemia intermediates [1, 2]. Various processes, the effects of increased hemolytic anemia, and chronic hypoxia on the liver, heart, skin, pancreas, and hypothalamus are well documented. Such problems should be considered in the normal treatment protocols for thalassemia patients, and follow-up should be done depending on the adverse effects they experience [3, 4]. Although medical data indicate that thromboembolic episodes occur more often, this problem has not been fully addressed or evaluated in patients with thalassemia intermedia, especially in cases of hypercoagulability in very young children, and the results of some investigations have been disputed [5–7]. Given the significance of thrombotic measures on patient survival and failure deduction of organs, including cardiovascular, lung, and brain, and the lack of evidence related to thrombotic status in patients, the present research aimed to evaluate assaying protein C and S levels in β-thalassemia intermedia [8].

The discovery of persistent hypercoagulability in thalassemia is supported by research into coagulation protein. The number of coagulation factors and their inhibitors, as well as fibrinolytic systems, in the blood varies greatly. Patients with thalassemia of different ethnic backgrounds were found to have a low level of coagulation inhibitors [9]. Proteins S and C are two plasma proteins that require vitamin K to function and when coupled, can serve as a spontaneous anticoagulant. Anticoagulant activity is shown by the preferential inactivation of factors VIIIa and Va. Decreased amounts of such proteins could be caused by oral anticoagulants, disseminated intravascular coagulation, liver disorder, and other medications. These three, and, in protein S case, nephrotic condition, pregnancy, lupus erythematosus, and particular hormones may all cause anomalies in such proteins [10].

The cluster of the β-thalassemia gene is observed on chromosome 11. In this cluster, along with the β globulin gene, other genes, including Gamma (Gg, Ag), Delta (δ), and Epsilon (ε), are observed. These genes are found in the fetus at various stages. The β gene is only found in adults. The gene (β, ββ) has two variants in normal humans. The mutation type determines the severity of the illness. Some β gene mutations lead to a full loss of protein generation (β0), while others (changes in the gene's regulatory region) lead to a decrease in protein generation (β+).

Both β/β0 and β/β+ have a secondary carrier known as β-thalassemia. Patients with the β+/β+ genotype had phenotypic levels that are moderate. Those people typically live normal lives, although blood transfusions may be necessary in the event of sickness or pregnancy, depending on the degree of the anemia.

Individuals with main β0/β0 and β0/β+ are affected by severe anemia. Their bodies have abnormal red blood cells. People have increased blood production in their bone marrow, changing the shape of their bones over time. Furthermore, these individuals require blood transfusions, which can lead to problems such as elevated iron levels. Heart failure and arrhythmia can both be caused by iron overload [11]. This study assessed the levels of protein C and S in β-thalassemia intermedia patients and connected them to various clinical and laboratory indicators to develop a preventative approach for individuals at risk of thromboembolic events.

## MATERIAL AND METHODS

This case-control study included 23 β-thalassemia intermedia patients who visited the Thalassemia Center in Erbil, Kurdistan, and 20 healthy people chosen as a control group, matched by gender and age.

Each patient was examined at the Thalassemia Center in Erbil. Age, sex, blood group, Rh, and residency were all recorded as demographic data. In addition, a complete clinical history was taken, including current infection, viral illness, splenectomy, and thrombosis history.

Patients' medical records were used to obtain hematological and biochemical information. The initial limited thromboplastin time, fibrinogen level, protein C, and protein S were measured in both case and control samples. Blood samples were taken from the patients and the control group prior to blood transfusion.

The protein C and S tests, liver function tests, and serum ferritin were all measured using the Aeskulisa protein C and S kit. A sandwich ELISA coated with a human protein-specific capture antibody was used for these experiments (BioTek ELX800).

Protein C concentrations in normal human plasma typically vary from 70 to 140%, whereas protein S concentrations range from 60 to 150%. Tables and graphs were used to show the results. Data were registered and analyzed using the Statistical Package for Social Sciences (SPSS) version 21. The means of continuous variables and the proportions of categorical were analyzed using Student t-test and Chi-square tests, respectively, and P-values less than 0.05 were regarded as significant.

## RESULTS

An important decrease in proteins C and S was observed in the patient group as compared with the control (89.35% (43–153) *vs*. 146% (75–210), P=0.041 and 94.43% (38–142) *vs*. 168.84% (82–196), P-value≤0.001, respectively) (Table 1, Figure 1 A–B). Protein C was significantly lower in older patients (>10 years) compared to younger ones (<10 years) and in patients with splenectomy compared to those without splenectomy (P=0.02 and 0.04, respectively) (Table 2). Protein S was significantly lower in patients with splenectomy compared to patients without splenectomy (P=0.04) (Table. 2). Even though this link did not reach statistically significant levels, it demonstrated significant relationships between protein C and S liver functions.

**Table 1:** Age, gender, and laboratory characteristics of the participants in the groups.

Data	Control group	Patients	P-value
Median of age	17.4	16.32	0.54
Patients under 10 years	7	8	0.65
Patients older than 10 years	13	15	0.45
Male	10	12	0.61
Female	10	11	0.61
Splenectomized	Zero	13	<0.001
Non-splenectomized	20	10	0.02
Mean of S. ferritin	88.5	1243	<0.001
Mean of protein C%	146%	89.35%	0.041
Mean protein S%	168.84%	94.43%	<0.001
SGPT U/l	26	58	0.09
SGOT U/l	18	63	0.04

**Figure 1A:**
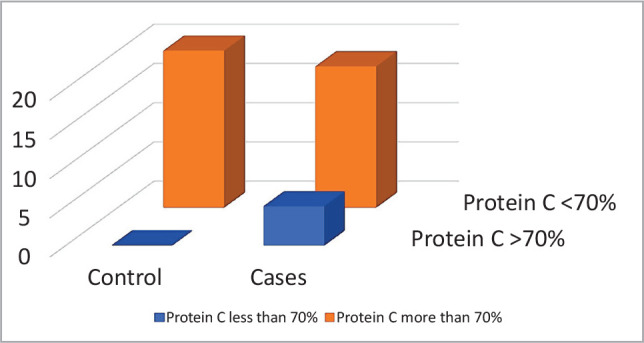
Normal and below normal protein C levels in patients and control group.

**Figure 1B:**
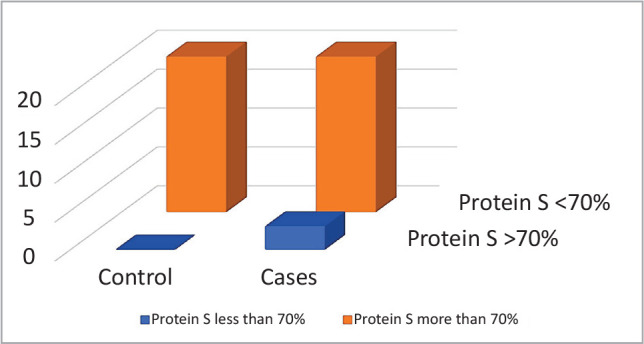
Normal and below normal protein S levels in patients and control group.

**Table 2:** Protein C and S levels in comparison to other clinical and laboratory parameters.

	No. of participants	Protein C	P-value	Protein S	P-value
**Gender**					
Male	12	99.5	0.52	85	0.15
Female	11	89.0		96	
**Age**					
<10	8	105.0	0.03	120.64	0.12
>10	15	82.75		113.40	
**Ferritin**					
<1000	7	99.5	0.50	128	0.46
>1000	16	87.0		102	
**Splenectomy**					
Yes	13	72	0.02	84	0.04
No	10	104		125	
**Liver function**					
Normal	7	104	0.15	98	0.06
Abnormal	16	67		61	

## DISCUSSION

One of the most recent complications in β-thalassemia patients is hypercoagulability, leading to severe morbidity and mortality [12]. This complication is not confined to β-thalassemia intermedia patients; activation of many hemostatic systems has also been demonstrated in persons with thalassemia major [12–14]. Even though transfusion and splenectomy ignorance are by far the most common causes of thalassemia, plenty of other causal variables have been proposed [15, 16].

This study examined the levels of natural anticoagulants (protein C and S) in β-thalassemia intermedia patients compared to healthy controls. In this investigation, only 5 and 3 patients had protein C and S levels lower than normal compared to healthy controls. Other research [14] reported similar findings, although with varying percentages of affected people, like Abosdera et al. [17], who discovered that 56 percent of thalassemia patients had lower protein C levels than normal.

The low amounts of naturally occurring anticoagulants in thalassemia patients were thought to be caused by a variety of factors. Because proteins C and S are formed in the liver, they are extremely susceptible to any synthetic function deficiency, which is prevalent in those patients due to a variety of offending factors, including hemosiderosis and viral hepatitis [10, 11, 14, 18, 19]. This was revealed by the statistically insignificant but evident connections between anticoagulant levels and liver function [serum glutamic-oxaloacetic transaminase (SGOT) and serum glutamic pyruvic transaminase (SGPT)]. Researchers believe liver dysfunction is not the main cause of their deficit [20]. Another cause for this is the adsorption of phospholipids negatively charged to the red blood cell (RBC) membrane, especially phosphatidylserine, which is abnormally present in the RBC external membrane of thalassemic patients [21]. Moreover, some authors thought that high level of anticoagulant consumption in thalassemia is due to continuous subclinical activation of the coagulation system, which is evidenced by increased thrombin-antithrombin (TAT) complexes levels found in thalassemia patients compared to normal persons [10, 22].

Proteins C and S were compared with clinical and laboratory aspects of diverse patients to determine the risk of having a hypercoagulable state. Protein C and S levels were considerably lower in the splenectomized group than in the non-splenectomized group. These results could be explained by the presence of procoagulants on the surface of RBCs and aberrant platelets, which are no longer cleared from the circulation following splenectomy, increasing protein C consumption to manage the hypercoagulable state [23, 24]. While some writers found similar results, other investigations did not [22, 25].

Protein C levels decreased more prominently in older patients. Musumeci et al. [26] observed similar results, with the lowest protein C values found in elder splenectomized individuals. Taher et al. [27] investigated the data of 8,860 thalassemia patients from Iran and the Mediterranean region and discovered that age was one of the risk variables for developing thromboembolic events. Given that the age of the study group was younger than the age for thromboembolic events development, it could explain why none of our patients suffered clinically overt thromboembolic events.

There were no significant variations in anticoagulant levels between men and women, indicating the same risk of developing thromboembolic events. Karami et al. [25] found lower levels in men; however other studies found no significant gender differences [27–29]. Further research is required to determine whether long-term usage of preventive anticoagulants is necessary to avoid subclinical thrombosis [6, 30]. The increased clinical and laboratory data on patients at risk of hypercoagulability support the development of a thrombosis prevention strategy.

## CONCLUSION

Proteins C and S (naturally occurring anticoagulants) levels were much lower in thalassemia patients, particularly in splenectomized and elderly patients. This result underlines the need for additional research and early preventative measures to prevent thromboembolic events in thalassemia patients.

## Personal thanks

I would like to express my great appreciation to Professor Ahmed Yassen Khudhair and Senior Doctor Mohammed Darwish for their help in evaluating and investigating the cases. In addition, I would like to thank Dr. Anne Salim, head of the laboratory at Nanakaly Hospital, where the investigations were achieved.
